# Lay Explanatory Models of Depression and Preferred Coping Strategies among Somali Refugees in Norway. A Mixed-Method Study

**DOI:** 10.3389/fpsyg.2016.01435

**Published:** 2016-09-22

**Authors:** Valeria Markova, Gro M. Sandal

**Affiliations:** ^1^Department of Pulmonology, Haukeland University HospitalBergen, Norway; ^2^Department of Psychosocial Science, University of BergenBergen, Norway

**Keywords:** depression, help-seeking, coping strategies, refugees, Somalia, mixed method, focus group

## Abstract

**Objective:** Refugees are at high risk of experiencing mental health problems due to trauma in their pasts and to acculturation stress as they settle in a new country. To develop efficient health services that meet the needs of refugees from different regions, an understanding is required of how they make sense of and prefer to cope with mental health problems. This study aims to investigate lay explanatory models of depression and preferred coping strategies among Somali refugees in Norway.

**Methods:** The study used a mixed-method design with a vignette describing a moderately depressed person based on ICD-10 criteria. Firstly, a survey study was performed among Somali refugees (*n* = 101). Respondents were asked to give advice to the vignette character and complete the Cross-Cultural Depression Coping Inventory and the General Help-Seeking Questionnaire. Secondly, focus group interviews (*n* = 10) were conducted separately with males and females to examine the relationship between the explanatory models of depression and the preferred coping strategies.

**Results:** The participants showed a strong preference for coping with depression by religious practices and reliance on family, friends, and their ethnic/religious community, rather than by seeking professional treatment from public health services (e.g., medical doctors, psychologists). Depressive symptoms were conceptualized as a problem related to cognition (thinking too much) and emotion (sadness), but not to biological mechanisms, and they were thought to result from spiritual possession, stress as a result of social isolation, and/or past trauma. Independently of time in exile, the participants showed a strong identification with their ethnic origin and associated values. Because participants emphasized the need to obey and follow the views of elders, fathers, and spiritual leaders, these authorities seemed to be “gatekeepers” for access to mental health services.

**Conclusion:** The results highlight that mental health programs for Somali refugees should actively involve the ethnic community, including spiritual leaders, in order to reach patients in need and to foster treatment compliance.

## Introduction

One of the largest refugee populations worldwide comes from Somalia. Because of the ongoing civil war, which has lasted since 1991, more than one million Somalis have fled to other countries in Africa, Europe, and North America (United Nations High Commissioner for Refugees, 2015). Immigrants and refugees, and particularly those coming from war zones, are at high risk of mental health problems due to factors such as trauma before and during their flight, acculturative stress, low socioeconomic status, social isolation, and feelings of powerlessness in the country of settlement (Dalgard et al., 2006; Lindert et al., 2009; Abebe et al., 2014). Research has documented that mental health problems are more prevalent among refugees than in the native population and other migrant populations (e.g., Bhurga, 2004; Lindert et al., 2009; Missinne and Bracke, 2012). Epidemiological evidence among Somali refugees is sparse and divergent, however (Bhui et al., 2006; Feyera et al., 2015) a survey study of Somali refugees in Norway reported a prevalence rate of 16% for anxiety and depression, compared to 9% for the general population (Blom, 2008). In Finland, the rate of moderate or severe depression was 21.1% among Somalis and 14.1% among native Finns (Mölsa et al., 2014). Bhui et al. (2006) found depression and anxiety to be present in 33.8% of Somali refugees residing in the United Kingdom.

The task of preventing, recognizing, and appropriately treating common mental health problems among refugees might be complicated because of differences in language, culture, patterns of seeking help, and ways of coping. Scholars have noted that it may be essential to present mental health care services in culturally sensitive ways in order to increase access to, use, and benefits of mental health care services (Ell et al., 2007; Wu et al., 2014), because beliefs about mental health among refugees often differ from the Western biomedical perspective on mental illness. Mental illness is hugely stigmatized in the Somali community, and access to psychiatric and psychological treatment is absent for the majority of the population. Until now, however, few studies have examined the understanding of mental health problems and preferred treatment and coping patterns among Somali refugees settled in Europe (Gladden, 2012). The aim of this study is to fill this gap in the literature. We focus on depression because of its high prevalence among refugees, and comorbidity with other common diseases in this population, such as anxiety and post-traumatic stress disorder (PTSD).

According to the World Health Organization, depression is a leading cause of disease burden worldwide (World Health Organization, 2013). Nevertheless, extensive research literature shows that there is wide variation across nations and ethnic groups in the way in which *depression* is explained and expressed (Hagmayer and Engelmann, 2014; Napier et al., 2014). How people understand the cause, manifestations, and treatment of illness has been referred to as *lay theories*, as opposed to the scientific models more frequently endorsed by professional caregivers in Western societies (see Furnham and Kirkcaldy, 2015). Linked to these theories are *explanatory models* defined as sets of ideas about episodes of disease that are held by patients and the practitioners involved in their treatment (Kleinman, 1980). Ideas about health and diseases are belief systems organized around concepts of causes (Knettel, 2016). The significance of health professionals paying attention to patients' explanatory models is highlighted by research showing that these belief systems are linked to a variety of responses, including attitudes (e.g., stigma) to compliance with treatment, to patient satisfaction, and to lifestyle changes aimed at managing diseases (Petrie and Weinman, 2006; Hagmayer and Engelmann, 2014).

The association between explanatory models and the use of health services is an area where further research is needed. Studies in several countries suggest that immigrants and refugees are less likely than their native-born counterparts to seek out or be referred to mental health services, even when they experience comparable or higher levels of distress (DeShaw, 2006; Dyhr et al., 2007; Sandvik et al., 2012; Norwegian Institute of Public Health, 2014). In Norway, where this study was carried out, Somali refugees are found to use acute psychiatric help or mental health specialists less frequently than the majority population, while they are more likely to seek help from emergency primary health care (Sandvik et al., 2012; Norwegian Institute of Public Health, 2014). Half of their contact with emergency primary health care was for non-specific pain (Sandvik et al., 2012). Research has documented differences in how patients from different cultures present symptoms of depression (Kuittinen et al., 2014). For example, a study conducted in Finland showed that older Somali refugees manifested more somatic-affective symptoms of depression than native Finns, whereas native Finns manifested more cognitive symptoms than the Somalis (Kuittinen et al., 2014). Such findings may provide insight into why immigrants from poorer countries tend to be given ill-defined diagnoses more often than native-born citizens. The diagnoses given are often related to musculoskeletal conditions, whereas diagnoses of mental disorders tend to be more infrequent (Grünfeld and Noreik, 1991; Sandvik et al., 2012). American research has shown that clinicians diagnose ethnic majority individuals with psychiatric illness more often than they do ethnic minority individuals exhibiting the same symptoms (Skaer et al., 2000), ostensibly believing the symptoms to be normative for the minority group (Pottick et al., 2007). Along the same lines, a study conducted in Great Britain found that Somali refugees suffering from anxiety and depression or psychosis were more likely to be on physical care medication than undergoing psychological treatment (Bhui et al., 2003a).

Disparities in help-seeking behavior and treatment between refugees and the native-born population have been attributed to language barriers (Wiking et al., 2004), healthcare providers' lack of cultural competence (Sandhu et al., 2013), and lack of knowledge about what services are available (Open Society Foundations, 2015). Somali refugees' mistrust of the biomedical health sector, which is sometimes the result of unfulfilled expectations of medical encounters, have been reported in studies conducted in several countries, including Sweden (Svenberg et al., 2011), the USA (Scuglic et al., 2007), and the Netherlands (Feldman et al., 2006). While the possible impact of all these factors is recognized, we argue that explanatory models about mental health problems play a vital role in the coping pattern observed among Somali refugees. Napier et al. (2014) explicates the association between culture and health, arguing that culture can be understood as not only habits and beliefs about perceived wellbeing, but also political, economic, legal, ethical, and moral practices and values. An understanding of the explanatory models of Somali refugees needs to consider characteristics of their culture of heritage. We focus here on culture at the level of nationality, which is common in the cross-cultural literature, while recognizing that nations are rarely homogeneous. According to Schwartz (2006), sub-Saharan cultures are characterized by an emphasis on embeddedness and hierarchy that implies expectancies of obedience, conformity, and group identification. Against this backdrop, we assume that Somali refugees adhere strongly to culturally shaped beliefs and practices as regards how they understand and cope with depression.

*Coping strategies* in this paper refers to the way in which people prefer to react to or deal with depression, including help-seeking behavior and preferred treatment. In general, *coping* refers to the thoughts and behaviors people use to manage the external and internal demands of stressful events (Lazarus and Folkman, 1984). Depression has been regarded as a reflection of hopelessness or helplessness due to unsuccessful coping (Levine and Ursin, 1991). However, depression itself is an adverse condition that individuals may deal with in different ways. Contemporary stress models emphasize that the initial appraisal of the situation normally directs the choice of coping strategies. Thus, the choice of strategies for dealing with mental health problems depends on the explanatory model (Karasz, 2005). Consistent with this theoretical prediction, an extensive literature review of studies of causal beliefs about depression in different cultural groups by Hagmayer and Engelmann (2014) showed that causal beliefs were closely linked to coping preferences. They categorized assumed causes into five categories: stress (externally caused), personality and psychological causes (e.g., thinking too much), biological causes (e.g., chemical imbalance), supernatural causes (e.g., witchcraft, god's will), and traditional causes (causes based on non-western medical theories, e.g., traditional Chinese medicine). In addition, coping strategies were classified into five categories: psychological treatment (e.g., psychotherapy), social support (i.e., non-professional support from family and friends), bio-medical treatment (e.g., antidepressant medication), religious (e.g., praying) or supernatural practices, and non-Western medicine or alternative treatment (e.g., yoga, herbs, healers). We adopt the same classifications in the present study.

Hagmayer and Engelmann (2014) noted that, for Western groups in particular, causal beliefs were clearly related to treatment preferences. These groups were most in favor of bio-medical treatment, followed by social support. Religious and supernatural practices came third, ahead of traditional treatment and psychotherapy. Those who believed more strongly in supernatural causes endorsed religion as a treatment more than others. However, people within the same cultural group may endorse more than one causal factor and have different views about efficient coping strategies, depending on the assumed causes. For instance, Okello and Ekblad (2006) investigated causal beliefs about depression among the Ganda people in Uganda. When witchcraft was suspected to be the cause, the help of traditional healers was sought, while Western medicine was preferred to address assumed somatic causes. Similarly, in the Somali context, people tend to endorse religious and supernatural treatment for mental disorders. According to a report from the World Health Organization (2010), the mainstay of mental health care in Somaliland is social support, followed by traditional and religious healers (mostly herbalists and faith healers). Mölsa et al. (2010) noted that, for Somalis in exile, traditional understandings, and practices relating to mental distress may change as a result of immigration and acculturation processes. However, many Somali refugees have limited prior experience of bio-medical treatment methods, because mental healthcare infrastructure is nonexistent in Somalia following the civil war (Leather et al., 2006; Syed Sheriff et al., 2011). Research suggests that many Somali refugees maintain traditional Somalian beliefs and practices about mental health problems even after they settle in a new country. For example, a study of Somali refugees living in Finland suggested that mental disorders tended to be seen as reflecting spiritual and/or social problems (Mölsa et al., 2010). Similarly, Carrol (2004) concluded that religion, supernatural practices, and alternative treatments seemed to carry more weight than bio-medical and psychological treatment among Somali refugees in the USA. A qualitative study showed that Somali participants drew strength from interdependence and the connection they felt to their social network or religious faith when experiencing mental distress (Jorden et al., 2009). Sources of social support for refugees may still differ from the support refugees could access in Somalia, since families may be separated during the immigration process and loved ones may have been lost through war (Smith, 2013).

To date, the literature on how Somali refugees tend to perceive and cope with mental health problems is limited. As noted by Gladden (2012), existing studies have been based on an explorative and qualitative approach, and they usually rely upon small samples comprising mostly male migrants. The present study tries to overcome these limitations by using a mixed-method design, in line with recommendations by other researchers (Bhui and Bhugra, 2002; Hagmayer and Engelmann, 2014). Firstly, we conducted a survey among Somali refugees to assess how they view the likelihood of seeking help from different sources when experiencing symptoms of depression, and their coping patterns in dealing with this condition. This is followed by focus-group interviews to elaborate on the results from the quantitative study and to gain a deeper understanding of how help-seeking and coping patterns are linked to etiological beliefs about depression. The term *refugee* in this paper refers to persons with legal residence who have come to Norway for protection, including those who have come through family reunification. Our study includes lay people of Somali origin rather than a clinical population. In this paper, lay people refer to persons who do not have specialized or professional knowledge of mental health disorders. The high prevalence of depression among Somali refugees suggests that a large proportion will either experience this disorder themselves or have to cope with family members who experience it. Research suggests that, particularly in communal cultures, family members will have a strong influence on mental health service utilization and choice of coping strategy (see references in Erdal et al., 2011). Thus, the views of lay people may be highly informative about how refugees experience and deal with mental health problems.

## Sub-study 1

### Methods

#### Participants

The sample consisted of Somali refugees above the age of 18 years living in Norway. A total of 101 respondents (response rate 33%) participated in the study. Six respondents were excluded from the data analysis because more than 80% of their data were missing. The final sample consisted of 95 respondents (44% women), 75 of whom completed the full questionnaire. Of the respondents, 34% were taking or had a university or college degree. More demographic information about the sample is provided in Table 1.

**Table 1:** **Demographic statistics for men and women—Sub-Study 1**.

	**Males *n* = 53**	**Females *n* = 42**	**Total *n* = 95**
Age	*M* = 28 *(SD* = 9.28)	*M* = 28 (*SD* = 7.35)	*M* = 28 (*SD* = 8.43)
Duration of stay (Years)	*M* = 9 (*SD* = 9.62)	*M* = 10 (*SD* = 8.24)	*M* = 10 (*SD* = 9.27)
**REASON FOR MIGRATION**			
Work/Studies	7%	0%	4%
Asylum seeker	57%	43%	51%
Family reunion	30%	55%	41%
I came with my parents	6%	2%	4%
**CURRENT SITUATION**			
Employed	25%	29%	27%
Student (including those attending a Norwegian language course)	60%	43%	53%
Unemployed	9%	10%	10%
Work at home	0%	7%	3%
Leave of absence	0%	5%	2%
Early retirement/disability benefit	0%	0%	0%
Other	4%	5%	4%
Missing	2%	1%	1%
In a relationship (married, living with a partner, long-term boyfriend or girlfriend	38%	50%	43%
Not in a relationship	62%	45%	55%
Missing	0%	5%	2%

#### Materials

The first part of the survey asked about demographic information, including age, sex, reason for migration, years of formal education, cause of immigration, and current employment situation. The second part of the survey consisted of a vignette, describing a person with symptoms of depression consistent with the criteria for a depressive episode in the International Classification of Diseases-10 (World Health Organization, 2011). The gender of the vignette character was matched to the respondent to facilitate identification. We used the same vignette that was used in a previous study on mental health conceptions among immigrants to Norway (Erdal et al., 2011). The vignette read as follows:

“*John/Ann is a 27-year-old waiter in a restaurant in Bergen. He/she was born in Oslo to parents who were restaurant owners, but has made Bergen his/her home for 5 years. In the last few weeks, he/she has been experiencing feelings of sadness every day. John/Ann's sadness has been continuous, and he/she cannot attribute it to any specific event or to the season. It is hard for him/her to go to work every day; he/she used to enjoy the company of his/her co-workers and working at the restaurant, but now he/she cannot find any pleasure in this. In fact, John/Ann has little interest in most activities that he/she once enjoyed. He/she is not married and lives alone, near his/her brother/sister. Usually, they enjoy going out together and with friends. But now he/she does not enjoy this anymore. John/Ann feels very guilty about feeling so sad, and feels that he/she has let down his/her brother/sister and friends. He/she has tried to change his/her work habits and start new hobbies to become motivated again, but he/she cannot concentrate on these tasks. Even his/her brother/sister has now commented that John/Ann gets distracted too easily and cannot make decisions. Since these problems began, John/Ann has been sleeping poorly every night; he/she has trouble falling asleep and often wakes up during the night. A few nights ago, as he/she lay awake, trying to fall asleep, John/Ann began to cry because he/she felt so helpless*.”

After reading the vignette, the respondents answered questions on two scales. The first measured help-seeking behavior and the second measured coping strategies.

*The General Help-Seeking Questionnaire* (GHSQ; Wilson et al., 2007) consists of 19 items describing different sources from whom to seek help. Each item was rated on a 6-point Likert scale (1 “very unlikely” to 6 “very likely”). The standard instruction “*If you were having [problem-type], how likely is it that you would seek help from the following people?*” (Wilson et al., 2007) was modified to: “*If you were feeling like Ann/John (gender matched), how likely is it that you would seek help from the following sources?*” In line with the recommendations of Wilson et al. (2007), we added items to fit the target group. Specifically, based on past research on how refugees cope with health-related problems (Carrol, 2004; Hagmayer and Engelmann, 2014), we included items referring to sources of help in the refugees' ethnic community (e.g., traditional healers, elders in the community, leaders in the ethnic community or from the same country) and alternative medicine (e.g., acupuncture, homeopathy). One source (the Norwegian Labor and Welfare Administration (NAV)) was added to adapt to the Norwegian context. In addition, an open space (with the heading “other”) was provided for participants to indicate other help-seeking sources that they would use and that were not listed.

*The Cross-Cultural Depression Coping Inventory* (CCD-CI) assesses how immigrants prefer to deal with depressive symptoms. An instrument used in a previous study (Erdal et al., 2011) was revised and extended to cover a broad range of coping strategies of relevance to refugees from different cultures. To avoid an ethnocentric bias in the coping behaviors listed, researchers from several disciplines (anthropology, social work, psychology) and people from many countries (e.g., Ghana, USA, Korea, China, Norway) were involved in generating relevant items. The survey was pilot tested on the target population and other immigrant groups to check whether the items were meaningful and unambiguous. Finally, the pool of items was reviewed by a panel of researchers to reduce overlapping items. The final version of the CCD-CI consists of 28 statements describing different ways in which the vignette character could deal with his/her depression (see **Table 3**). The respondents were asked to indicate their agreement with each statement on 6-point Likert scales (1, “strongly disagree”; 6, “strongly agree”).

#### Procedure

The study was approved by the Local Regional Committee for Medical Research Ethics and Norwegian Social Science Data Services (NSD). In line with the Helsinki declaration, participants signed a declaration of consent and received written information about the study. They were informed that individual information would be kept confidential and told how data would be stored and reported. The survey was administered in English and Norwegian. Translations of the instruments were completed using a traditional forward and back translation, comparing versions to maximize technical, semantic, content, and conceptual equivalence (Flaherty et al., 1988). The survey was distributed and collected on paper (*n* = 33) or online (*n* = 68). No monetary rewards were offered. The paper version was administered to two samples of Somali refugees in Bergen, the second largest city in Norway. One sample consisted of participants in Norwegian language courses (each with ~20 students) at the largest school for adult immigrants, while the second consisted of visitors at the only Somali café in Bergen. A native Somali explained the project in Somali, and the participants had an opportunity to ask questions in Somali about the project or to clarify the content and meaning of the items. As for the online survey, nine Somali immigrant organizations were contacted by email. Two of them agreed to give the researchers access to their Facebook group (about 1000 members in total) and sent individual invitations to group members on behalf of the researchers, asking them to participate in the study on Facebook. Only members above the age of 18 years were contacted. Participants received only one invitation. Participants gave their consent to participate in the online survey by pressing the “Next” button after reading information about the project.

### Results

#### Preferred coping strategies

In terms of sources of help (GHSQ) as shown in Table 2, the most notable findings (all over the mean of 4.0) are the emphasis on social support (e.g., parents, intimate partner, friends, and other relatives/family members) and the religious community. Eleven respondents made use of the response alternative, “other.” These included faith (*N* = 5; for example: *Allah (God*); *read Quran and pray to Allah for help, because I think that it is only God who can help me with everything*) and friends and family (*N* = 3). The following comments were added by the remaining three respondents: “*Someone that I trust can help me or he has gone through what I go through at the moment*,” “*My only friend (ME)*,” and “*One that knows Somali and who is a psychologist*.”

**Table 2:** **Descriptive statistics of items in the GHSQ**.

**Item**	**Men**	**Women**	**Total**	**95% C.I**.
	***M* (*SD*)**	***M* (*SD*)**	***M* (*SD*)**	
(1) Parent	4.9 (1.8)	5.0 (1.5)	4.9 (1.6)	4.58–5.22
(2) Intimate Partner (e.g., girlfriend, boyfriend, husband, wife)	4.2 (1.9)	4.9 (1.4)	4.5 (1.7)	4.16–4.84
(3) Friends	4.2 (1.6)	4.7 (1.6)	4.5 (1.6)	4.18–4.82
(4) Religious leader (e.g., priest, rabbi, chaplain, mullah)	4.4 (1.9)	4.4 (1.7)	4.4 (1.8)	4.04–4.76
(5) Other relative/Family member	4.4 (1.7)	3.7 (1.9)	4.1 (1.8)	3.74–4.46
(6) Medical doctor/General Practitioner (GP)	3.4 (1.9)	4.5 (1.5)	3.9 (1.7)	3.56–4.24
(7) Elders in my community	3.4 (1.8)	2.5 (1.8)	3.0 (1.8)	2.64–3.36
(8) Other people in my ethnic community or from the same country as me	3.3 (1.7)	2.5 (1.7)	2.9 (1.7)	2.56–3.24
(9) Psychiatrist/psychologist	3.2 (1.9)	2.6 (1.7)	2.9 (1.8)	2.54–3.26
(10) Internet forums	2.8 (1.9)	2.5 (1.7)	2.6 (1.8)	2.24–2.96
(11) A work colleague	2.7 (1.8)	2.4 (1.5)	2.6 (1.6)	2.28–2.92
(12) Leaders in my ethnic community or from the same country as me	2.7 (1.7)	2.3 (1.5)	2.5 (1.6)	2.18–2.82
(13) Alternative medicine (e.g., acupuncturist, homeopath)	2.5 (1.8)	2.4 (1.6)	2.4 (1.7)	2.06–2.74
(14) My manager or human resource staff at my workplace	2.7 (1.6)	2.0 (1.6)	2.3 (1.6)	1.98–2.62
(15) Social worker/NAV	2.5 (1.8)	1.8 (1.2)	2.2 (1.6)	1.88–2.52
(16) Traditional healer	2.3 (1.6)	1.8 (1.4)	2.2 (1.5)	1.9–2.5
(17) I would not seek help from anyone	2.2 (1.6)	1.9 (1.4)	2.1 (1.5)	1.8–2.4
(18) I would seek help from someone not listed above	2.2 (1.8)	1.8 (1.4)	2.0 (1.6)	1.68–2.32
(19) Phone helpline	2.3 (1.8)	1.8 (1.3)	2.0 (1.6)	1.68–2.32

*Value varied from 1, very unlikely to 6, very likely*.

The most preferred coping strategies (mean score above 5.0), assessed using CCD-CI, as shown in Table 3, were religious practice (e.g., reconciliation with God, personal prayer, or getting someone to pray for him/her), social support (e.g., find a partner), and alternative treatment (e.g., get more rest, reflect on his/her life, express emotions, spend more time in nature, engage in leisure activities, physical exercise, avoid thinking too much). The least preferred coping strategies (mean score below 3.0) were the use of medication, use of alcohol or other drugs, and passive coping strategies, including “there is nothing wrong with John/Ann,” “not tell anyone about his/her feelings,” “blame someone else and be ashamed.”

**Table 3:** **Descriptive statistics of items in the CCD-CI**.

**Item**	**Men**	**Women**	**Total**	**95% C.I**.
	***M (SD)***	***M (SD)***	***M (SD)***	
(1) …reconcile him-/herself with God	5.9 (1.4)	5.6 (1.9)	5.7 (1.7)	5.36–6.04
(2) …get more rest	5.4 (1.4)	5.7 (1.4)	5.6 (1.4)	5.32–5.88
(3) …reflect on his/her life	5.6 (1.4)	5.6 (1.7)	5.6 (1.5)	5.3–5.9
(4) …express his/her emotions	5.2 (1.9)	5.9 (1.1)	5.6 (1.6)	5.28–5.92
(5) …spend more time in nature	5.5 (2)	5.6 (1.6)	5.5 (1.8)	5.14–5.86
(6) …engage in leisure time activity to get his/her mind off the situation	5.8 (1.8)	5.1 (1.7)	5.5 (1.8)	5.14–5.86
(7) …avoid thinking too much	5.3 (1.9)	4.8 (2.2)	5.1 (2)	4.7–5.5
(8) …find a partner	5.2 (2.2)	5.1 (1.9)	5.1 (2.1)	4.68–5.52
(9) …pray or get someone to pray for him/her	5.6 (1.8)	5.3 (1.8)	5.4 (1.8)	5.04–5.76
(10) …reassess his/her life situation	5.7 (1.4)	5 (1.8)	5.3 (1.6)	4.98–5.62
(11) …get more physical exercise	5.4 (2)	5.2 (1.6)	5.3 (1.8)	4.94–5.66
(12) …get married	4.7 (2.5)	4.2 (2.5)	4.5 (2.5)	4–5
(13) …start practicing yoga or meditate	4.5 (2.2)	4.4 (1.9)	4.4 (2.0)	4–4.8
(14) …get help to reconsider his/her diet	4.6 (2.1)	3.8 (2)	4.2 (2)	3.8–4.6
(15)…keep him-/herself busy with work	4.3 (2.3)	4.1 (2.1)	4.2 (2.2)	3.76–4.64
(16) …get a pet	4 (2.2)	3.1 (2)	3.6 (2.1)	3.18–4.02
(17) …talk courage into him-/herself	3.2 (2.2)	3.9 (2.2)	3.6 (2.2)	3.16–4.04
(18) …has no reason to be sad	3.9 (2.3)	2.6 (2)	3.3 (2.2)	2.86–3.74
(19) …get help to find out if he/she is a victim of malevolent witchcraft or evil spirit	3.1 (2.1)	3.3 (2.1)	3.2 (2.1)	2.78–3.62
(20) …stay at home until he/she gets better	3.1 (2.4)	3.3 (2.1)	3.2 (2.2)	2.76–3.64
(21) …start using herbs and natural remedies	2.7 (2)	3.6 (2.1)	3.1 (2.1)	2.68–3.52
(22) …does not need to do anything, it is just something that will go away by itself	3.3 (2.1)	2.7 (1.9)	3 (2)	2.6–3.4
(23) …use medication	3.4 (2.3)	2.2 (1.7)	2.8 (2.1)	2.38–3.22
(24) There is nothing wrong with John/Ann	3.3 (2.2)	2 (1.7)	2.7 (2)	2.3–3.1
(25) …not tell anyone about his/her feelings	1.8 (1.7)	2.5 (2.1)	2.1 (1.9)	1.72–2.48
(26) …blame someone else	1.9 (1.4)	2.1 (1.8)	2 (1.6)	1.68–2.32
(27) …be ashamed	2.0 (1.7)	1.8 (1.6)	1.9 (1.6)	1.58–2.22
(28) …use alcohol or other drugs	1.6 (1.6)	1 (0.2)	1.3 (1.2)	1.06–1.54

*(…), Ann/John should/needs to. Value varied from 1, strongly disagree to 6, strongly agree*.

Overall, the confidence intervals are less than 1 unit on the raw response scale, which means that the intervals are rather precisely located and that the population means are unlikely to differ by more than 1 unit from their sample values. Moreover, relatively few intervals include the extreme scale points (1 and 6). Only the first item goes beyond 6, while no items are below 1, which makes the occurrence of floor effects very unlikely and ceiling effects also rather unlikely.

## Sub-study 2

### Methods

#### Participants and procedure

Two focus group interviews were conducted. The groups were divided by gender and consisted of four men and six women. Table 4 shows the demographic characteristics of the participants. The interviewees were recruited from the Introductory Program for Refugees, which is a public program that is compulsory for recent refugees above the age of 18 who are granted a residence permit in Norway. The participants were chosen to produce maximum variation with respect to age, education level, work experience, and marital status. Most of the participants received an invitation to participate in and information about the project 1 week prior to the event.

**Table 4:** **Demographic characteristics of the participants—Sub-Study 2**.

**Participants**	**Age**	**Residence time in Norway**	**Education**	**Family status**
Man 1	25–30	1–2 years	Not finished primary school	Lives alone
Man 2	50–55	4–5 years	Primary school	Lives with family, several children
Man 3	30–35	2–3 years	University	Married, lives alone
Man 4	25–30	1–3 years	Primary school	Lives alone
Woman 1	40–45	1–2 years	Not finished primary school	Lives with family, one child
Woman 2	55–60	3 years	Not finished primary school	Married, lives alone
Woman 3	25–30	<1 year	University	Married
Woman 4	25–30	2–3 years	Primary school	Married
Woman 5	25–30	1–2 years	High school	Married, lives alone
Woman 6	30–35	2–3 years	Unknown	Lives alone

#### Focus group interviews

Two interviewers (the authors) conducted the interviews. Because the participants had limited Norwegian language skills, licensed translators of Somali origin were used. Group discussions lasted for ~2 h. The same vignette was used as in the survey. The gender of the vignette character was matched to the respondents' genders, and Somali names were used (Ali and Nora) to facilitate identification. Prior to the interviews, participants consented to being filmed, and they were informed about their rights as research subjects, including that they could withdraw from the interview at any time or refuse to answer questions. They were also informed that their responses would be treated confidentially. They signed a declaration of consent, in which this information was given. The participants were encouraged to maintain confidentiality about information gained about each other during the interview. Refreshments, such as tea, fruit, and cakes, were served during the interview.

One of the interviewers read the vignette to the participants before asking questions for discussion. The same interview guide was followed for both interviews. The main questions were as follows: “Does Ali/Nora have a problem?,” “What are the most efficient ways of dealing with his/her condition?,” and “What are the likely reasons for the condition?.” The questions followed an interview guide that was developed based on theoretical considerations and on exploratory, in-depth interviews conducted during the preparatory stages of the project. The interviewer maintained a high level of control over the discussion, introducing general issues and probing or interjecting to ensure that the groups covered all the essential points, that all participants were active, and that the conversation was focused. Considerable latitude was given to permit free discussion of issues, unsolicited opinions, and unexpected responses.

#### Analysis

Videotapes from the focus group interviews were transcribed verbatim into Norwegian, masking the identity of participants. In addition, facial, non-verbal communication cues were transcribed. Data were analyzed in accordance with the principles of Template Analysis (King, 2007), which means that key topics are defined in advance. However, these topics can be modified, dispensed with, or augmented if those defined a priori do not prove to be useful or appropriate to the actual data examined. The transcripts were coded using NVIVO 10 (NVivo Qualitative Data Analysis Software., 2012). The classification of causal beliefs and coping strategies by Hagmayer and Engelmann (2014) served as a starting point for a priori categories used to analyze the data. The coding frame was then elaborated and modified as new themes and subthemes emerged in the course of the analysis. For the superordinate theme “causes,” the “supernatural causes” category was merged with the “traditional causes” category. Religious causes/explanations were also included in this category, which was labeled “supernatural, religious, or traditional causes.” For the superordinate theme “coping strategies,” the “non-Western,” and “alternative treatment” categories were combined to form one category labeled “palliative coping.” It was defined as looking for diversions and occupying oneself with other things in order not to think about the problem, for example, by trying to feel better by relaxing or exercising (Sandal et al., 1999).

### Results

#### Explanatory model of depression and possible causes

##### Explanatory model of depression

All the participants were able to associate the symptoms of the vignette character with either someone they knew or themselves. The depressed condition described in the case was explained by participants as “illness of thoughts” or something spiritual inside the person that has to be taken out. This (spiritual) entity was described as having both cognitive and emotional components, but no biological basis. Man 2 made the following comment:“*[making a gesture from the heart and as if he wants to symbolize that weight must be taken off his heart] We have a proverb [in Somalia] that says that, if we have things that we keep to ourselves, the thing may hold you back also. That means that having a problem and keeping it to yourself is a problem in itself. It will only increase the problem. So to speak out and speak to others, it is of real help!*.”

Choice of coping strategy depended on their understanding of the underlying causes, as illustrated by the following quote from Man 3: “*What I would do first…[in] the first place, I would have talked with him and I would ask why, what had happened to him, I would have a conversation with him and find out.…So after I have received information from him, I would have found out and assessed what assistance I could offer him…problems that you may experience, there might be different causes [for them]. You may have family problems, and this [disorder] has something to do with the family, or he may have experienced war and been subjected to serious situations. The other things that can cause sadness and stuff [in the] described situation, [that could be] for example, the use of drugs…a lot of drugs*.” Both groups discussed the most likely explanation for the condition of the vignette character and appropriate coping strategies, and agreed upon the importance of considering psychological and supernatural, religious, and traditional causes. While stress was also mentioned as a possible cause, there were differences among participants about the kind of stressors that could cause depression. Biological explanations were briefly mentioned, but only in the male group.

##### Personality/psychological causes

Most of the participants attributed the depressive symptoms of the vignette character to his/her family situation, specifically to not being married and living alone. Being single meant that it was difficult to receive support from others, and it could also lead to feelings of loneliness: Man 1 commented: “*Loneliness is the reason why he has such a difficult time. If you live entirely by yourself all alone, one thinks very much about different things, there are lots of things that come into your head [points to his head]*.”Several of the participants linked the situation of the vignette character to their experience of loneliness when coming to Norway. Man 3 said: “*I can only mention that, when I was in my home country, I lived with my wife, I also lived with my other family. And I had a job to go to. But what I have right now…I can only attend Norwegian courses and go home. It is only me who lives here…alone…so it can also be difficult*.”Man 4 added: “*When I was sick in my home country, there everybody was with me, even my grandparents, all my siblings…Relatives…They would come to me and show me their support…They would show me that they care. By showing they cared about me, because of that, you become relieved in a way, you feel like they are sharing with you, this pest, and this illness. But here, even today, I feel ill, but there is no one sharing with me. Everything is for me alone*.”

In Somali culture, family is a broad term, including not just nuclear family members, but also uncles, aunts, cousins, and more distant relatives, sometimes even people who are not biologically related, such as members of the same clan/community. Participants in the interviews mentioned that, when one person experiences a problem, the family will meet and discuss the nature of the problem. In this way, the community decides the reason for the problem. Parents and elderly members of the community are especially influential in this process. The following quote from Man 4 illustrates how the perception of cause and coping choices were interlinked: “*I can only mention that*…*if one gets sick in Somalia*; *primarily one has to talk about his illness to his parents*. *So parents will find out about what kind of illness their children have and if they find out it is a psychological problem, mental illness, they find someone who is something like a healer, one who knows the Quran, an institution where they can treat that, if they mmm* * *find that it is a disease that the body has received*…*that needs medical care, they seek help from a doctor*. *It depends on what kind of problem you have*.” The latter quote is in line with the results from the survey. It indicates that parents' views strongly influence the use of mental health services, as well as which treatments are deemed appropriate.

The authority and respect expected to be shown to parents and the elderly was described as sometimes frustrating by the younger participants, who noted that it collides with the more egalitarian values of Norwegian culture. Participants in both groups mentioned that younger Somalis in Norway may expect more freedom and autonomy than older members of the community are ready to give them. Man 3 said that this gap, caused by cultural difference, may prevent parties from seeing and understanding each other: “*In particular, I can say that, if the parents come from Somalia or Ethiopia or other African countries [talking about the parents of the person in the case], they bring with them the solidarity, the collective…identity…so it might be difficult for them to see…and understand…[that] their children have grown up here and bring with them a different culture. Their children, who grow up here, these kids have with them the Norwegian culture as well. They live for themselves and they keep more to themselves. So what we can see here [talking about the case], we can see that he has grown up here, he has the Norwegian culture with him as well [in addition to the immigrant culture]*.” This situation may cause frustration for young people, as illustrated by the following quote from Woman 3: “*We have a culture where we respect the elderly very much, parents are very important. Sometimes we don't want to, but we respect what they have to say, yes. Even, if we don't agree with them. Because of respect, we agree with them even if we don't agree. This can also create sadness*.”

##### Supernatural, religious or traditional causes

The significance of religion (Islam) to mental health beliefs and coping preferences was strongly emphasized in the interviews. Being a “*bad*” Muslim, Jinn possession, or both, could be reasons for feeling like the vignette character. The following quote is from one of the oldest participants, Man 2: “*Research shows that people who experience stress and psychiatric disorders, it is mostly people who don't believe in anything*.” Following this statement, the participants explained that Muslims who don't follow the guidelines described in the Quran (regarding smoking, drinking, etc.) have a higher chance of “getting” mental illness. *One* quote from a member of the male group illustrates this: “*We believe that good Muslims don't get psychiatric disorders and stress like that [as described in the vignette]*.”

Until the end of the interview, neither of the groups discussed religious beliefs, especially the folk belief that mental health problems can be caused by Jinns. When the topic was introduced, the translator for the female group asked: “[Participants have a lively discussion, then the translator asks them to pause] *I can tell you one thing.…Have you heard about Jinn?* [Interpreter speaks slowly]…*Do you know what Jinn means*?” The participants explained that belief in Jinns is strong in the Somali community and that Jinns are often perceived as being responsible for mental illnesses. Jinns have the ability to possess and to take over people's minds and bodies. They can take different physical forms, and they have free wills like human beings. Jinns are normally invisible in the human realm, while humans appear clearly to Jinns. The participants described several ways in which humans may become possessed by Jinns, for example by walking in areas inhabited by these spirits (e.g., mountains or by the sea). The possibility that the vignette character is possessed by a Jinn was discussed by both groups. They concluded that this could only be determined by an imam or a healer, who also possesses the power to expel the spirit.

##### Stress (externally caused)

Participants recognized that depression could result from stress and trauma before and during the flight from Somalia. Trauma, such as rape, witnessing violence, war crimes, or losing a close friend or relative, were mentioned as experiences that could predispose a person to experiencing depressive symptoms. However, the participants differed in their willingness to discuss difficulties in the past. Older participants, in particular, were less active when talking about trauma. Woman 5 commented: “*It's like past…[points at her head] something is haunting her, something that made her sad happened to her, and now it is haunting her and it is making her sad. I don't know, maybe something very bad happened in her childhood, something in the past and it is haunting her, sometimes it stays for a while, sometimes she forgets. Maybe she is scared or…Maybe she thinks that it is going to come back …Because, I also had it very painful and could not sleep and stuff. Then I went to the doctor, and he said, you are not sick, but maybe you have something that you think about. When I found out [what was bothering me] I became completely healthy. But maybe she has something in her mind…that she has not seen yet*.” Woman 1 interrupted and said: “*Or maybe she had a boyfriend and they were going to get married and then he said no [most participants laugh]*.”

##### Biological causes

Substance abuse, especially of khat, was mentioned by both groups as a possible reason for the condition of the vignette character. Khat is a green-leafed shrub that has been chewed for centuries by people who live in the Horn of Africa and the Arabian Peninsula. Chewing khat was described as common among men in the Somali communities, also in Norway. Members of both groups stressed that they would not use khat themselves, but that they knew people who did. None of the respondents mentioned other physiological mechanisms (medical problems) as possible reasons for the condition of the vignette character.

#### Coping strategies

Coping strategies were grouped into five categories: social support, religious, or supernatural practices, palliative coping, bio-medical treatment, and psychological treatment. Because many participants found it difficult to conclude about the main cause of the depressed condition of the vignette character, they emphasized the necessity of considering several coping strategies simultaneously.

##### Social support

Virtually all participants described the importance of seeking social support when trying to cope with symptoms of depression. Talking about the problem was thought to be helpful in terms of releasing emotions. Moreover, seeking the company of others was seen as important to distract the depressed person from excessive worries and intrusive thoughts. In line with the results from the survey, the most important sources of social support were considered to be parents, family authorities, or a spouse. However, since many Somalis do not have their family or intimate partner in Norway, participants also talked about the need to draw on other people in their ethnic community, e.g., friends or other Muslims. Several of the female participants stressed the importance of making active use of all available social resources, including neighbors and work colleagues. Woman 6 commented: “*Instead of just sitting alone, behind locked doors, it is much better to go to family, friends, or, not least, to neighbors, and talk. You talk and get better, you become healthier*.” As the vignette character was described as being single, it was emphasized that he/she needed to get married. Finding a spouse was thought to stimulate new thoughts and perspectives on life, to give them other problems to think about (e.g., children) and someone to lean on for emotional support. These factors were seen as important by participants, regardless of age or gender. Man 1 commented: “*I think that if he marries, he will get somebody he can be with and then he will not need to feel lonely as he gets somebody to be with, somebody to share with. Happiness and all that*.” Woman 1 said: “*Maybe if she marries, perhaps with somebody…finds a man, her life will change, they are together, she becomes pregnant…maybe she gets a baby. Then it becomes a completely different life, really, in a way…If someone becomes like that in Somalia [referring to the vignette character], they must marry at once. So they get better!*”

The collective responsibility of the Somalis in supporting members of their community was highlighted. Support could entail advice about treatment and coping behavior, financial aid for seeking treatment abroad, finding a suitable partner to marry, religious guidance, and praying. This is illustrated by the following comments from Man 1: “*Since he is 27 years old, somebody should find him somebody to marry! If he marries, he will not be alone anymore. [Most participants nudge at each other]…*” Man 2: “*If the family don't have enough money to send him for treatment, the extended family and friends-community will help…Family will pray for him, and ask others to pray for him*.”

##### Religious or supernatural practices

Even though religious coping behaviors were addressed late in the discussions, they proved to be seen as key coping strategies. Participants emphasized that Islam obliges Muslims to care about each other as they all are members of one religious community. They also talked about their belief that life on Earth is a test that everyone must go through before they are rewarded with a better life with Allah. This was illustrated by the following comment by Man 4: “*I think that for us Muslims, if I, for example, get this kind of problem, this type of problem [referring to the case], and my friends come to visit me, the first thing they would start with, the first thing they would recommend me to do, they would recommend me to look after and to take care of my religion and follow it and practice [being a good] Muslim in a way, and they would also give me advice, advice…they would also say that; it is a short life, another world exists, and we work toward another life*.”

In line with the quantitative data, the most important religious practice was reading or listening to the Quran, either alone or together with family, followed by seeking advice and guidance from a religious leader or a professional healer. Quran readings were believed to help to cure all illnesses, as illustrated by the following comment from one of the older participants, Woman 1: “*Somebody should read from the Quran for her! Because the Quran can cure everything! All illnesses. Yes!*” The participants differed in their opinions about whether the condition of the vignette character was severe enough to engage an imam. There was also a discussion, specifically between female participants, about whether reading or listening to the Quran should be tried before medical treatment was sought, concurrently with medical treatment, or following medical treatment if the treatment was ineffective.

Several participants talked about acquaintances suffering from similar symptoms to the vignette character who had been dissatisfied with the treatment they received from Western health practitioners, and the positive effects once traditional “treatments” were used. Man 3 commented: “*I know a girl here in Norway, and they went to different doctors in Norway, but nothing helped, then I and several people from our community recommended her family to read from the Quran, and that helped*.” Participants said that many Somali people preferred to return to Somalia or other African countries for treatment. In Somalia, people would contact a traditional healer in addition to an imam if a problem such as that described in the vignette arose. Healers in Somalia could work independently or alongside general practitioners or an imam, with practices often including herbal medications, prayer rituals, and fire burning (touching the skin with a heated stick). The comments made by Man 1 exemplify how a visit to Somalia could also have other positive effects on depressive symptoms: “*I also experienced earlier that many people from the diaspora, they return from Somalia, they get this kind of psychological problem, stress and stuff, and then the reason for that is, the people were accustomed to living in a very large family, but suddenly, when they came to Europe, they were completely alone, they had nothing between work and home, they had no others. So when they get a problem like that in the diaspora, they return to Somalia. So the first treatment they receive, and what we also experienced when we were there, is treatment with the Quran*.”

##### Palliative coping

Various physical activities (e.g., yoga and walking), traveling, and getting more rest were considered effective means of temporarily reducing depressive symptoms. This is in line with the results from the survey. Man 4 commented: “*He lived in Oslo, alone and without family, and then for him it was important to take time off from work and rest, and change the climate and that can help! Yes. That can help*.”Stress-reducing activities needed to be congruent with being a Muslim. For example, one of the younger female participants, Woman 6, noted that she could not exercise in a health studio because she was a Muslim, but she did yoga at home and went for long walks. Another woman commented that walking long distances improved her sleep: “*Yes for me, it can help, physical activity! Because, for me, before I could not go to sleep early, not before one o'clock, I was not tired, I started exercising, finish at 20, home at 21, then I am ready to sleep*.”Khat chewing was also mentioned as a possible way of reducing stress. Even though participants mentioned that khat is commonly used by Somali males, they highlighted that they would not recommend it due to its serious side-effects.

##### Bio-medical treatment

Bio-medical treatment refers to visiting a medical doctor and to the use of medication. The survey data showed that women were more positive toward visiting medical doctors, although taking medication for relieving depression was not endorsed. These findings were in line with data from the interviews. While several female participants told about positive experiences with medical doctors, the older participants in both groups, in particular, were more skeptical. The possible benefits of taking medication were briefly mentioned. If medical doctors were contacted, concrete advice and solutions were expected, as illustrated by the following comment from Woman 5: “*I had [referring to the problem as described in the vignette], but not thinking…I just had a little bit of pain in my back [points to her back] I was very sad, very…I didn't want to go to school, when I went to the doctor, and then he told me, you don't have any problems, you are just stressed. I had some family problems. And he told me that, if you continue like this, you will develop bigger problems. It's best that you start doing physical exercise. I cannot give you any medicine, but I can only send you to a psychologist. I didn't go to a psychologist, but I did like what my doctor told me, I went to do physical training…All the pain that I had here [points to her back again], I had a lot of pain, and it's gone [claps her hands together], I just did what my doctor told me. Now I am happy*.” Most participants agreed that they would contact a doctor if they had suffered from a more severe condition than the vignette character. One women with a medical background said: “*In Somalian culture, actually, if you have stomach pain or something like that, you can go to the doctor, but with Nora's problems [the vignette character], then they don't go to the doctor so much. They will often try in another way. They will go to an imam or a Sheikh so they will read the Quran for her. Yes. So if it gets worse in a way, then one can go to a psychologist*.”

##### Psychological treatment

Psychological treatment, referring to the use of a psychologist or other mental health worker, was briefly mentioned early in one of the interviews. However, their ideas about psychologists seemed vague, and psychologists were sometimes confused with medical doctors. Like medical doctors, psychologists were expected to provide concrete solutions that would effectively cure the depressed person. Woman 3 commented: “*For me.…If she goes to a psychologist, she can tell all about how she feels. Her sadness…that she cries during the night. Things like that. So, maybe the psychologist can find a solution*.” When she was asked about what she thought a psychologist could help her with, she answered: “*A psychologist…or a doctor, doctor, doctor have learned how to treat in a way…A psychologist understands your soul. Yes. So he can find a solution. Yes!*”

## Final discussion

The aim of this mixed-method vignette study was to identify lay explanatory models of depression among Somali refugees in Norway. The results showed that religion and social relationships carried great weight, both in relation to etiological beliefs and views about efficient coping behavior. Both the survey and the interviews suggested that these refugees were likely to try to cope with depression through religious practices and reliance on family, friends, and the ethnic/religious community, rather than through professional treatment from public mental health services.

### Etiological beliefs and implications for treatment

Past research has shown that seeking professional mental health treatment is relatively rare among Somali refugees compared to the majority population. The findings from this study suggest that how Somali refugees interpret and treat depression is distinct from the common understanding among people in Western countries, who are apt to view depression as a mental illness requiring professional treatment (Karasz, 2005; Hagmayer and Engelmann, 2014). Depressive symptoms tended to be interpreted by our respondents as an “illness of thoughts” (“thinking too much”), with cognitive and emotional components but no biological basis. Depression, as described in the vignette, was not perceived as a physical or medical disease requiring professional treatment; rather, it tended to be seen as a condition primarily caused by supernatural or religious influences (e.g., being a “bad Muslim,” possession), the social situation, and/or as an emotional reaction to difficult life situations (e.g., loneliness and isolation). Similar findings were reported in a study carried out in Uganda, where depressive symptoms (without psychotic features) were also referred to as “illness of thoughts” and associated with thinking too much (Okello and Ekblad, 2006).

The interpretations of the causes of the depression had implications for how our respondents thought the condition should be managed. Engagement in leisure activities and spending time with family (or other people) were viewed as efficient ways of coping (interviews and survey), and as ways of diverting attention from the problem and stopping rumination (interview). Religious practices were suggested when the suspected cause was religious/spiritual. The view that the condition of the vignette character could be the result of spiritual causes or possession by an evil spirit is consistent with the traditional belief system of Somali culture (Ryan, 2007; Mölsa et al., 2010). El-Islam (2008) noted that belief in the existence of Jinns may prevent patients and family members from recognizing medical or psychiatric problems. According to Bentley and Owens (2008), many Somalis believe in religion as medicine, more than in interventions by a doctor or multidisciplinary team. For the respondents in the present study, the first line of healthcare treatment was religious practices: reconciliation with Allah (survey) and reading the Quran (interview). These findings are consistent with the literature and emphasize the significance of religion in helping Somali Muslim immigrants and refugees to cope with difficult circumstances (Whittaker, 2005; Mölsä et al., 2016). Some participants in the interviews indicated that medical doctors were not perceived as useful because they have no power over the spirits, which, according to folk belief, may be responsible for mental illnesses. Everyday practicing of Islam could also be considered a palliative coping strategy, because it provides relief and comfort that help to alleviate concerns and worries.

Aside from religion, the life situation of the vignette character was the most frequently discussed possible cause of his or her depressed condition. In particular, the vignette character's situation as unmarried and living apart from his or her family was highlighted. On a more general level, loneliness due to leaving behind their social network in Somalia was mentioned as one of the main reasons why Somali refugees develop symptoms of depression. Some participants indicated that depression is not as common in Somalia as it is in Norway, because people are embedded in a tight and supportive social network of family and clan members. However, trauma prior to or during flight and acculturation challenges were also mentioned as factors that could explain the condition. When symptoms are viewed as emotional reactions to life events and situations, this is referred to as a *situational model of depression*, which is found to be a common belief system in traditional societies and minority communities in the West (Patel, 1995; Karasz, 2005; Cabassa et al., 2008). This belief system implies that the source of the disorder is not within the individual but rather outside him/her—it is the context that requires “adjustment.” Studies show that a situational model is often associated with negative attitudes toward professional treatment (Jorm et al., 2000; Lauber et al., 2003). According to Karasz (2005), the Western perspective tends to view emotions as internal, often biological and, above all, a feature of individuals rather than related to situations, relationships, or moral positions. Divergent ontological and epistemological systems may yield divergent and even conflicting approaches to diseases in clinical practice. Western bio-medical guidelines prescribe antidepressants and psychotherapy as the most effective treatment for depression (World Health Organization, 2012; National Institute of Mental Health, 2013), and lay people from Western countries tend to share this view (Schomerus et al., 2012).

In the survey, seeking help from medical doctors was ranked below seeking help from family, friends, and religious leaders, a finding that is in line with the perspectives of the participants in the interviews. This suggests that mental health treatment in the context of the social group and religion may create more acceptance and compliance among Somalis. Individual therapy seems to be at odds with the collectivistic approach of Somali culture, a factor highlighted by the participants in the focus group interviews.

### Ethnic identity

Many Somalis in exile identify strongly with their ethnic group (Jorden et al., 2009; Lambo, 2012) and with being Muslim (Brons, 2001; Mölsa et al., 2010; Lambo, 2012). In the present study, the significance of culturally related beliefs and norms was reflected in the causal attributions of the depressive symptoms and in what was regarded as effective coping behavior. Somali culture organizes relations of interdependency in role-based hierarchical terms. In terms of coping behavior, the impact of a strong ethnic identity could be observed in several areas. Firstly, both the interviews and the survey data showed that the refugees chose to turn to members of their ethnic community for support and guidance before seeking help from public health services. This is in line with other research (Colic-Peisker and Tilbury, 2003). In the interviews, several participants emphasized that Somalis in the diaspora had a strong responsibility for looking after each other; this was also because of the bonds to the same religion (Islam). The importance of showing obedience to the views of the elderly, parents, and religious leaders was emphasized in the interviews. Thus, these authorities may act as “gatekeepers” for access to local mental health services, particularly for women and youth. Secondly, ethnic/religious identity seemed to some extent to limit the coping behavior deemed appropriate; for example, several females in the present study preferred individual exercise, such as yoga at home or walking rather than attending mixed-gender classes at gyms, because that was not appropriate according to Islam. Thirdly, several of the participants in the interviews suggested that traveling back to Africa could be an effective means of alleviating depressive symptoms. This was due to factors such as climate and the opportunity to interact with family and other clan members. Visiting Africa was also thought to give access to better traditional healers and mental health care services than are available in Europe. The latter aspect is consistent with past studies (Feldman et al., 2006; Gerritsen et al., 2006; Svenberg et al., 2011), which have suggested that low confidence in European health care and a wish for a “second opinion” lead many Somalis to seek medical advice and treatment in other countries. According to Tiilikainen and her colleagues (Tiilikainen and Koehn, 2011; Tiilikainen, 2012), transnational care is an important resource for Somali migrants. They noted that traditional healers in Somalia provide migrants with explanations and alternatives, in particular in the area of mental health and chronic diseases where medical diagnoses may be difficult to accept.

Kuo (2011) noted the existence of divergent coping patterns among immigrants with varying degrees of acculturation, and specifically that less acculturated cohorts preferred collective coping and avoidance coping methods. That the respondents in our study tended to prefer to approach mental health problems according to the belief systems of their culture of origin rather than using local health services is consistent with many Somali refugees being poorly integrated into Norwegian society. At the group level, they rank lower on psychosocial parameters such as employment and income (Østby, 2016), education, housing, and literacy than other ethnic groups (Ihle and Haider, 2001; Klepp, 2002; Engebrigtsen, 2004). Segregation, or rather the concentration of minorities in particular areas, has been observed in several European countries (Open Society Foundations, 2015). Somalis are found to be at risk of discrimination (Open Society Foundations, 2015), which is a further barrier to integration and may increase their experience of being “outsiders.” According to Fangen (2006), these features make Somali refugees in Norway vulnerable to feeling humiliated, and they may react by distancing themselves, leading to a reorientation to their own traditions and culture, and to living a life on the margins of Norwegian society. This perspective may also explain observations from the present study. That respondents in this study preferred to rely on their ethnic network to deal with depression may reflect that they feel isolated from mainstream society. They may also know little about local health services. There is a risk that this coping pattern could result in late treatment, if any is sought at all (Bhui et al., 2003b; McCrone et al., 2005).

## Limitations

The combination of a survey and focus group interviews enabled us to elicit rich contextual information that is rarely reported in the literature. Nonetheless, some methodological considerations should be borne in mind when interpreting the data. The data reported are cross-sectional. The study only focused on assessing causal beliefs and ideas about effective coping. Thus, we did not consider the potential effectiveness of different coping behaviors in terms of relieving depressive symptoms. The study leaves this important topic open for future research. The present study uses a vignette methodology. Participants in both interview groups readily recognized the symptoms described in the vignette, and no one seemed to have any difficulty interpreting them and recommending strategies for coping. However, we cannot conclude whether the responses of our subjects can be generalized to how they would have reacted personally in the vignette character's situation. The possibility of drawing inferences about the impact of culture on the explanatory model of our respondents is also limited by the lack of a native Norwegian (or Western) comparison group. Cultural differences in explanatory models may vary according to the severity of the depressed condition. In Somali culture, there is no gray area for mental disease; you are either sane, or insane (Nwaneri et al., 1999). Only the latter conditions are considered worth seeking medical/professional attention for Njenga et al. (2012). Thus there is no continuum of mental diseases. As the vignette character in this study suffered from a mild to moderate depression, he/she might have fallen outside the demarcation line of being perceived as “insane.”

Future studies may like to consider comparing the responses of a clinical sample to those of lay people. Methodologically speaking, longitudinal studies measuring and tracking how migrants' coping behaviors and mental health evolve across different phases of their acculturation process are desirable. Moreover, while individual variations were noted, our focus was on similarities in participants' perspectives rather than differences. Future research should more systematically address how differences might be related to individual factors, such as age, gender, education, pre-acculturation status, and acculturation.

Potential bias relating to the data collection methods and procedures also needs to be pointed out. In the interviews, the differences in the cultural backgrounds of the interviewers and the informants could have resulted in misunderstandings. The informants' trust in the interviewers and other participants, as well as feelings of shame and anxiety about the mental health problems themselves, may have influenced their motivation to express their thoughts and opinions. The responses of the survey participants could be biased by factors such as social desirability, lack of familiarity with questionnaires, and illiteracy. Nonetheless, the validity of the results is supported by the high consistency between the quantitative (survey) and qualitative (interview) data, and by correspondence with past research. Furthermore, resource persons from the Somali community were consulted prior to data collection and again later regarding interpretation of the results.

The reader should be mindful that the study was conducted in a Norwegian context and that it might not be possible to extrapolate the results to refugees living in other countries, because possible coping behaviors and help-seeking sources are contingent on the environment and structural resources (e.g., community or mental health services). The response rate for the survey is relatively low, but comparable to other studies among minority populations (Perez et al., 2011; Abbot and Compton, 2014). Nonetheless, one should be mindful that the results may not be generalizable to a more heterogeneous sample of Somali refugees in Norway. Compared to the larger population of Somali refugees in Norway, the employment rate was higher among the participants in the survey and the majority of the respondents were relatively young. Participation in the survey also required language and reading skills that many Somali refugees do not have, in particular those with shorter residence time in Norway.

## Implications and conclusion

The purpose of the current study was to explore how causal beliefs about depression are related to the choice of coping strategies among Somali refugees living in Norway. Despite its exploratory nature, the key strength of this study is its combination of quantitative and qualitative methodologies and the variation within our samples in terms of residence time. Consistent results emerged across the two sets of data. Taken together, the findings suggest that many Somali refugees continue to adhere strongly to causal beliefs and coping patterns from their culture of origin. The ethnic community influences which coping strategies are viewed as effective and acceptable, and it offers resources in the form of support and guidance. These results have implications for clinical practice. Establishing working alliances between mental health caregivers in the country of settlement, the Somali ethnic community, and religious/spiritual authorities might be critical in relation to reaching individuals in need and to their acceptance of and compliance with treatment. Equally important is the need for health professionals to discuss an explanatory model for the disease with the patients prior to diagnosis and treatment. For example, Dein and Illaiee (2013) noted that, since Western health professionals tend to be unfamiliar with the attribution of psychiatric symptoms to Jinns, diagnosis may prove challenging, especially when the patient–physician meeting is already impeded by language problems and cultural differences. Guthrie et al. (2016) showed that belief in Jinns and spirit possession may result in Somali patients being misdiagnosed by Western health professionals. According to other researchers (Khalifa et al., 2011; Dein and Illaiee, 2013; Lim et al., 2015), understanding the belief system and introducing a spiritual dimension to therapy may increase the efficacy of treatment among ethnic groups who are predominantly Muslim. Given the influx of immigrants to many European countries, ethnically tailored treatment programs may be integral to eliminating health care inequalities and providing high-quality patient care for all members of the population.

## Author contributions

Both authors have been active in the study design, data collection, analysis, and manuscript writing.

## Funding

The study was funded by the Western Norway Regional Health Authority (project number 911834).

### Conflict of interest statement

The authors declare that the research was conducted in the absence of any commercial or financial relationships that could be construed as a potential conflict of interest.

## References

[B1] Abbot O. Compton G. (2014). Counting and estimating hard-to-survey populations in the 2011 census, in Hard-to-Survey Populations, eds Tourangeau R. Edwards B. Johnson T. P. Wolter K. M. Bates N. (Cambridge: Cambridge University Press), 58–81.

[B2] Abebe D. S. Lien L. Hjelde K. H. (2014). What we know and don‘t know about mental health problems among immigrants in Norway. J. Immigr. Minor. Health 16, 60–67. 10.1007/s10903-012-9745-9 23117694

[B3] Bentley J. A. Owens C. W (2008). Somali Refugee Mental Health Cultural Profile. Harborview Medical Center's Ethnic Medicine Website. Available online at: https://ethnomed.org/clinical/mental-health/somali-refugee-mental-health-cultural-profile (Accessed January 10, 2016).

[B4] Bhui K. Abdi A. Abdi M. Pereira S. Dualeh M. Robertson D.. (2003b). Traumatic events, migration characteristics and psychiatric symptoms among Somali refugees. Soc. Psychiatry Psychiatr. Epidemiol. 38, 35–43. 10.1007/s00127-003-0596-5 12563557

[B5] Bhui K. Bhugra D. (2002). Explanatory models for mental distress: implications for clinical practice and research. Br. J. Psychiatry 181, 6–7. 10.1192/bjp.181.1.6 12091256

[B6] Bhui K. Craig T. Mohamud S. Warfa N. Stansfeld S. A. Thornicroft G.. (2006). Mental disorders among Somali refugees. Developing culturally appropriate measures and assessing socio-cultural risk factors. Soc. Psychiatry Psychiatr. Epidemiol. 41, 400–408. 10.1007/s00127-006-0043-5 16520881

[B7] Bhui K. Stansfeld S. A Hull S. Priebe S. Mole F. Feder G. (2003a). Ethnic variations in pathways to and use of specialist mental health services in the UK. Br. J. Psychiatry 182, 105–116. 10.1192/bjp.182.2.105 12562737

[B8] Bhurga D. (2004). Migration and mental health. Acta Psychiatry Scand. 109, 243–258. 10.1046/j.0001-690X.2003.00246.x 15008797

[B9] Blom S. (2008). Innvandreres Helse 2005/2006 [Immigrant Health 2005/2006], 35. Available online at: http://www.ssb.no/emner/00/02/rapp_200835/rapp_200835.pdf.

[B10] Brons M. H. (2001). Society, Security, Sovereignty and the State in Somalia: From Statelessness to Statelessness? Groningen: International Books.

[B11] Cabassa L. J. Hansen M. C. Palinkas L. A. Ell K. (2008). Azùcar y nervivos: explanatory models and treatment experiences of Hispanic with diabetes and depression. Soc. Sci. Med. 66, 2413–2424. 10.1016/j.socscimed.2008.01.054 18339466 PMC2475593

[B12] Carrol J. K. (2004). Murug, Waali, and Gini: expressions of distress in refugees from Somalia. J. Clin. Psychiatry 6, 119–125. 10.4088/pcc.v06n0303 15361926 PMC474735

[B13] Colic-Peisker V. Tilbury F. (2003). “Active” and “Passive” Resettlement: The influence of support services and refugees‘ own resources on resettlement style. Int. Migr. 41, 61–91. 10.1111/j.0020-7985.2003.00261.x

[B14] Dalgard O. S. Thapa S. B. Hauff E. McCubbin M. Syed H. R. (2006). Immigration, lack of control and psychological distress: findings from the Oslo Health Study. Scand. J. Psychol. 47, 551–558. 10.1111/j.1467-9450.2006.00546.x 17107504

[B15] Dein S. Illaiee A. S. (2013). Jinn and mental health: looking at jinn possession in modern psychiatric practice. Psychiatry 37, 290–293. 10.1192/pb.bp.113.042721

[B16] DeShaw P. J. (2006). Use of the emergency department by Somali immigrants and refugees. Minn. Med. 89, 42–45. 16967884

[B17] Dyhr L. Andersen J. S. Engholm G. (2007). The pattern of contact with general practice and casualty departments of immigrants and non-immigrants in Copenhagen, Denmark. Dan. Med. Bull. 54, 226–229. 17850728

[B18] El-Islam M. F. (2008). Arab culture and mental health care. Transcult. Psychiatry 45, 671–682. 10.1177/1363461508100788 19091731

[B19] Ell K. Quon B. Quinn D. I. Dwight-Johnson M. Wells A. Lee P. J.. (2007). Improving treatment of depression among low-income patients with cancer: the design of the ADAPt-C study. Gen. Hosp. Psychiatry 29, 223–231. 10.1016/j.genhosppsych.2007.01.005 17484939 PMC1868447

[B20] Engebrigtsen A. (2004). Somaliere i eksil i Norge. En Kartlegging av Erfaringer Fra Fem Kommuner Og Åtte Bydeler I OSLO [Somalis in Exile in Norway. A Survey of Experiences from Five Municipalities and Eight Districts in Oslo]. Oslo: NOVA.

[B21] Erdal K. Singh N. Tardif A. (2011). Attitudes about depression and its treatment among mental health professionals, lay persons and immigrants and refugees in Norway. J. Affect. Disord. 133, 481–488. 10.1016/j.jad.2011.04.038 21620476

[B22] Fangen K. (2006). Humiliation experienced by Somali Refugees in Norway. J. Refugee Stud. 19, 69–93. 10.1093/jrs/fej001

[B23] Feldman T. Bensing J. M. De Ruijter A. Boeije H. R. (2006). Somali Refugees' experiences with their general practitioners: frames of reference and critical episodes. Int. J. Migr. Health Soc. Care 2, 28–40. 10.1108/17479894200600025

[B24] Feyera F. Mihretie G. Bedaso A. Gedle D. Kumera G. (2015). Prevalence of depression and associated factors among Somali refugee at melkadida camp, southeast Ethiopia: a cross-sectional study. BMC Psychiatry 15:171. 10.1186/s12888-015-0539-1 26204950 PMC4513619

[B25] Flaherty J. A. Gaviria F. M. Pathak D. Mitchell T. Wintrob R. Richman J. A.. (1988). Developing instruments for cross-cultural psychiatric research. J. Nerv. Ment. Dis. 176, 257–263. 10.1097/00005053-198805000-00001 3367140

[B26] Furnham A. Kirkcaldy B. (2015). Lay people's knowledge of mental and physical illness, in Promoting Psychological Well-Being in Children and Families, ed Kirkcaldy B. (New York, NY: Palgrave MacMillan), 14–32.

[B27] Gerritsen A. A. Bramsen I. Deville W. van Willigen L. H. Hovens J. E. van der Ploeg H. M. (2006). Use of health care services by Afghan, Iranian, and Somali refugees amd asylum seekers living in the Netherlands. Eur. J. Public Health 16, 394–399. 10.1093/eurpub/ckl046 16672251

[B28] Gladden J. (2012). The coping skills of East African refugees: a literature review. Refugee Survey Q. 31, 177–196. 10.1093/rsq/hds009

[B29] Grünfeld B. Noreik K. (1991). Uførepensjonering blant innvandrere i Oslo [Disability pension among immigrants in Oslo]. Tidsskrift for den Norske lœgeforening 111, 1147–1150. 2024268

[B30] Guthrie E. Abraham S. Nawaz S. (2016). Process of determining the value of belief about jinn possession and whether or not they are a result of mental illness. BMJ Case Rep. 2016:bcr2015214005. 10.1007/s00127-016-1198-3 26838303 PMC4746541

[B31] Hagmayer Y. Engelmann N. (2014). Causal beliefs about depression in different cultural groups-what do cognitive psychological theories of causal learning and reasoning predict? Front. Psychol. 5:1303. 10.3389/fpsyg.2014.01303 25505432 PMC4243491

[B32] Ihle R. Haider M. (2001). Somaliaprosjektet. Om Bosetting av Somaliere i 12 Kommuner På Vestlandet [Somalia Project. The Resettlement of Somalis in 12 Municipalities in Western Norway]. Utlendingsdirektoratet: Regionkontor vest.

[B33] Jorden S. Matheson, K. Anisman H. (2009). Supportive and unsupportive social interactions in relation to cultural adaptation and psychological distress among Somali refugees to collective or personal traumas. J. Cross Cult. Psychol. 40, 853–874. 10.1177/002202210933918226419721

[B34] Jorm A. F. Christensen H. Medway J. Korten A. E. Jacomb P. A. Rodgers B. (2000). Public belief systems about the helpfulness of interventions for depression: associations with history of depression and professional help-seeking. Soc. Psychiatry Psychiatr. Epidemiol. 35, 211–219. 10.1007/s001270050230 10941996

[B35] Karasz A. (2005). Cultural differences in conceptual models of depression. Soc. Sci. Med. 60, 1625–1635. 10.1016/j.socscimed.2004.08.011 15652693

[B36] Khalifa N. Hardie T. Latif S. Jamil I. Walker D. M. (2011). Beliefs about Jinn, black magic and the evil eye among Muslims: age, gender and first language influences. Int. J. Cult Mental Health 4, 68–77. 10.1080/17542863.2010.503051

[B37] King N. (2007). Template Analysis. Available online at: http://www.hud.ac.uk/hhs/research/template-analysis/what-is-template-analysis/

[B38] Kleinman A. (1980). Patients and Healers in The Context of Culture. Berkely, CA: University of California Press.

[B39] Klepp I. (2002). Ein Diskursiv Analyse av Relasjonar Mellom Somaliske Familiar og Lokalbefolkninga i ein Vestnorsk Bygdeby [A Discursive Analysis of Relationships Between Somali Families and the Local Population in a West Norwegian Town]. Hovedoppgave, Institutt for antropologi, Universitetet i Bergen.

[B40] Knettel B. A. (2016). Exploring diverse mental illness attributions in a multinational sample: A mixed-methods survey of scholars in international psychology. Int. Perspect. Psychol. 5, 128–140. 10.1037/ipp0000048

[B41] Kuittinen S. Punamäki R. L. Mölsä M. Saarni S. I. Tiilikainen M. Honkasalo M. L. (2014). Depressive symptoms and their psychosocial correlates among older somali refugees and native finns. J. Cross Cult. Psychol. 45, 1434–1452. 10.1177/0022022114543519

[B42] Kuo B. C. H. (2011). Culture's consequences on coping. Theories, evidences, and dimensionalities. J. Cross Cult. Psychol. 42, 1084–1100. 10.1177/0022022110381126

[B43] Lambo I. (2012). In the shelter of each other: notions of home and belonging amongst somali refugees in Nairobi, in New Issues in Refugee Research. Policy Development and Evaluation Service (UNHCR the UN refugee Agency). Available online at: www.unhcr.org/4face3d09.pdf

[B44] Lauber C. Nordt C. Falcato L. Rossler W. (2003). Do people recognize mental illness? Factors influencing mental health literacy. Eur. Arch. Psychiatry Clin. Neurosci. 2532, 48–251. 10.1007/s00406-003-0439-0 14504994

[B45] Lazarus R. S. Folkman S. (1984). Stress, Appraisal, and Coping. New York, NY: Springer Publishing Company.

[B46] Leather A. Ismail E. A. Ali R. Abdi Y. A. Abby M. H. Gulaid S. A.. (2006). Working together to rebuild health care in post-conflict Somaliland. Lancet 368, 1119–1125. 10.1016/S0140-6736(06)69047-8 16997666

[B47] Levine S. Ursin H. (1991). What is stress?, in Stress: Neurobiology and Neuroendocrinology, ed Brown M. R., (New York, NY: Marcel Dekkar), 3–21.

[B48] Lim A. Hoek H. W. Blom J. D. (2015). The attribution of psychotic symptoms to jinn in Islamic patients. Transcult. Psychiatry 52, 18–32. 10.1177/1363461514543146 25080427

[B49] Lindert J. von Ehrenstein O. S. Priebe S. Mielck A. Brähler E. (2009). Depression and anxiety in labor migrants and refugees - A systematic review and meta-analysis. Soc. Sci. Med. 69, 246–257. 10.1016/j.socscimed.2009.04.032 19539414

[B50] McCrone P. Bhui K. Craig T. Mohamud S. Warfa N. Stansfeld S.. (2005). Mental health needs, service use and costs among Somali refugees in the UK. Acta Psychiatry Scand. 111, 351–357. 10.1111/j.1600-0447.2004.00494.x 15819728

[B51] Missinne S. Bracke P. (2012). Depressive symptoms among immigrants and ethnic minorities: a population based study in 23 European countries. Soc. Psychiatry Psychiatr. Epidemiol. 47, 97–109. 10.1007/s00127-010-0321-0 21110000

[B52] Mölsa M. E. Hjelde K. H. Tiilikainen M. (2010). Changing conceptions of mental distress among Somalis in Finland. Transcult. Psychiatry 47, 276–300. 10.1177/1363461510368914 20603389

[B53] Mölsä M. Kuittinen S. Tiilikainen M. Honkasalo M. L. Punamäki R. L. (2016). Mental health among older refugees: the role of trauma, discrimination, and religiousness. Aging Ment. Health. 1–9. 10.1080/13607863.2016.1165183. [Epub ahead of print]. 27080403

[B54] Mölsa M. Punamäki R. L. Saarni S. I. Tiilikainen M. Kuittinen S. Honkasalo M. L. (2014). Mental and somatic health and pre- and post-migration factors among older Somali refugees in Finland. Transcult. Psychiatry 51, 499–525. 10.1177/1363461514526630 24648488

[B55] Napier A. D. Ancarno C. Butler B. Calabrese J. Chater A. Chatterjee H.. (2014). Culture and health. Lancet 384, 1607–1639. 10.1016/S0140-6736(14)61603-2 25443490

[B56] National Institute of Mental Health (2013). Depression. U.S. Department of Health and Human Services. Available online at: http://www.nimh.nih.gov/health/publications/depression-easy-to-read/index.shtml

[B57] Njenga F. G. Kigamwa P. Nguithi A. (2012). Culture and Mental Health: a comprehensive textbook. Psychiatry in East Africa, in Culture and Mental Health. A Comprehensive Textbook, eds Bhui K. Bhurga D. B (Boca Raton, FL: CRC Press), 160–169.

[B58] Norwegian Institute of Public Health (2014). Helse i Innvandrerbefolkningen [Health in Immigrantpopulation]. (Accessed August, 2016), Available online at: https://www.fhi.no/nettpub/hin/helse-i-ulike-befolkningsgrupper/helse-i-innvandrerbefolkningen—fo/psykisk-helse

[B59] NVivo Qualitative Data Analysis Software (2012). QSR International Pty Ltd. Version 10. Los Angeles, CA.

[B60] Nwaneri M. O. Barnes N. Adair R. (1999). Healthcare access for Somali refugees: view of patients, doctors, nurses. Am. J. Health Behav. 23, 286–287. 10.5993/AJHB.23.4.6

[B61] Okello E. S. Ekblad S. (2006). Lay concepts of depression among the Baganda of Uganda: a pilot study. Transcult. Psychiatry 43, 287–313. 10.1177/1363461506064871 16893877

[B62] Open Society Foundations (2015). Somalis in European Cities - Overview. Available online at: https://www.opensocietyfoundations.org/reports/somalis-european-cities-overview

[B63] Østby L. (2016). Refugees in Norway. Available online at: https://www.ssb.no/en/befolkning/artikler-og-publikasjoner/refugees-in-norway.

[B64] Patel V. (1995). Explanatory models of mental illness in sub-Saharan Africa. Soc. Sci. Med. 40, 1291–1298. 10.1016/0277-9536(94)00231-H 7610434

[B65] Perez D. F. Nie J. X. Ardern C. I. Radhu N. Ritvo P. (2011). Impact of participant incentives and direct and snowball sampling on survey response rate in an ethnically diverse community: results from a pilot study of physical activity and the built environment. J. Immigr. Minor. Health 15, 207–214. 10.1007/s10903-011-9525-y 21932003

[B66] Petrie K. J. Weinman J. (2006). Why illness perceptions matter. Clin. Med. 6, 536–539. 10.7861/clinmedicine.6-6-536 17228551 PMC4952762

[B67] Pottick K. J. Kirk S. A. Hsieh D. K. Tian X. (2007). Judging mental disorders in youths: effects on client, clinician, and contextual differences. J. Consult. Clin. Psychol. 75, 1–8. 10.1037/0022-006X.75.1.1 17295558

[B68] Ryan J. (2007). Going “Walli” and having “Jinni”: Exploring Somali Expressions of Psychological Distress and Approaches to Treatment. New Zealand: Department of Psychology; The University of Waikato.

[B69] Sandal G. M. Endersen I. M. Vaernes R. Ursin H. (1999). Personality and coping strategies during submarine missions. Milit. Psychol. 11, 381–404. 10.1207/s15327876mp1104_3 11543156

[B70] Sandhu S. Bjerre N. V. Dauvrin M. Dias S. Gaddini A. Greacen T.. (2013). Experiences with treating immigrants: a qualitative study in mental health services across 16 European countries. Soc. Psychiatry Psychiatr. Epidemiol. 48, 105–116. 10.1007/s00127-012-0528-3 22714866

[B71] Sandvik H. Hunskaar S. Diaz E. (2012). Immigrants‘ use of emergency primary health care in Norway: a registry-based observational study. BMC Health Serv. Res. 12:308. 10.1186/1472-6963-12-308 22958343 PMC3471038

[B72] Schomerus G. Schwahn C. Holzinger A. Corrigan P. W. Grabe H. J. Carta M. G.. (2012). Evolution of public attitudes about mental illness: a systematic review and meta-analysis. Acta Psychiatr. Scand. 125, 440–452. 10.1111/j.1600-0447.2012.01826.x 22242976

[B73] Schwartz S. H. (2006). A theory of cultural value orientations: explications and applications. Comparat. Sociol. 5, 137–182. 10.1163/156913306778667357

[B74] Scuglic D. I. Alarcòn R. D. Lapeyre A. C. III Williams M. D. Logan K. M. (2007). When the poetry no longer rhymes: mental health issues among somali immigrants in the USA. Transcult. Psychiatry 44, 581–595. 10.1177/1363461507083899 18089640

[B75] Skaer T. L. Sclar D. A. Robison L. M. Galin R. S. (2000). Trends in the rate of depressive illness and use of antidepressant pharmacotherapy by ethnicity/race: an assessment of office-based visits in the United States, 1992-1997. Clin. Ther. 22, 1575–1589. 10.1016/S0149-2918(00)83055-6 11192148

[B76] Smith Y. J. (2013). We all Bantu – we have each other: preservation of social capital strengths during forced migration. J. Occup. Sci. 20, 173–184. 10.1080/14427591.2013.786647

[B77] Svenberg K. Skott C. Lepp M. (2011). Ambiguous expectations and reduced confidence: experience of Somali refugees encountering Swedish health care. J. Refugee Stud. 24, 690–705. 10.1093/jrs/fer026

[B78] Syed Sheriff R. J. Reggi M. Mohamed A. Haibe F. Whitwell S. Jenkins R. (2011). Mental health in Somalia. Int. Psychiatry 8, 89–91. PMC6735037 31508100

[B79] Tiilikainen M. (2012). It's just like the internet: transnational healing practices between Somaliland and the Somali Diaspora, in Medicine, Mobility, and Power in Global Africa. Transnational Health and Healing, eds Dilger H. Kane A. Langwick S. A. (Indiana: Indiana University Press), 271–293.

[B80] Tiilikainen M. Koehn P. H. (2011). Transforming the boundaries of health care: insights from Somali migrants. Med. Anthropol. 30, 518–544. 10.1080/01459740.2011.577288 21916683

[B81] United Nations High Commissioner for Refugees (2015). UNHCR Statistical Yearbook 2014, 14th Edn. Available online at: http://www.unhcr.org/statistical-yearbooks.html

[B82] Whittaker S. (2005). An exploration of psychological well-being with young Somali refugee and asylum-seeker women. Clin. Child Psychol. Psychiatry 10, 177–196. 10.1177/1359104505051210

[B83] Wiking E. Johansson S. E. Sundquist J. (2004). Ethnicity, acculturation, and self reported health. A population based study among immigrants from Poland, Turkey, and Iran in Sweden. J. Epidemiol. Community Health 58, 574–582. 10.1136/jech.2003.011387 15194719 PMC1732838

[B84] Wilson C. J. Deane F. P. Ciarrochi J. Rickwood D. (2007). Measuring help-seeking intentions: properties of the general help seeking questionnaire. Can. J. Counsel. Psychother. 39, 15–28.

[B85] World Health Organization (2010). A Situation Analysis of Mental Health in Somalia. Available online at: http://www.who.int/hac/crises/som/somalia_mental_health/en/

[B86] World Health Organization (2011). International Classification of Diseases: Mood Disorders: depressive Episode, 10th Edn. Available online at: http://apps.who.int/classifications/apps/icd/icd10online/

[B87] World Health Organization (2012). Depression in Europe. World Health Organization Regional office for Europe Available online at: http://www.euro.who.int/en/countries/latvia/news/news/2012/10/depression-in-europe

[B88] World Health Organization (2013). Mental Health Action Plan 2013-2020. Available online at: http://www.who.int/mental_health/publications/action_plan/en/

[B89] Wu B. Jin H. Vidyanti I. Lee P. J. Ell K. Wu S. (2014). Collaborative depression care among latino patients in diabetes disease management, Los Angeles, 2011-2013. Prevent. Chro. Dis. 11, E148. 10.5888/pcd11.140081 25167093 PMC4149319

